# Supplementary material for: In Situ Polymerization of Linseed Oil-Based Composite Film: Enhancement of Mechanical and Water Barrier Properties by the Incorporation of Cinnamaldehyde and Organoclay
Source: Molecules. 2022 Nov 21;27(22):8089. doi: 10.3390/molecules27228089 (PMC9699561; doi:10.3390/molecules27228089)
Supplement: Supplementary file 1 [file molecules-27-08089-s001.zip › molecules-1967968-supplementary.pdf]

Supplementary Information

In situ Polymerization of Linseed Oil-based Composite Film: Enhancement of Mechanical and Water Barrier Properties by the Incorporation of Cinnamaldehyde and Organoclay

Rim Guesmi [1], Nasreddine Benbettaieb [2,*], Mohamed Ramzi Ben Romdhane [3,4], Thouraya Barhoumi-Slimi [1,4] and Ali Assifaoui [2,*]

[1] Department of Chemistry, Laboratory of Structural (Bio)Organic Chemistry and Polymers. Faculty of Sciences of Tunis, University of Tunis El Manar, Tunis, Tunisia;
[2] UMR PAM, University of Burgundy, Institut Agro, Dijon, France
[3] Laboratory of Composite Materials and Clay Minerals, National Center of Researches in Material Sciences (CNRSM), Hammam lif,Tunisia
[4] High Institute of Environmental Science and Technology, Technopark of Borj Cedria, University of Carthage,Hammam-lif, Tunisia
* Correspondence: nasreddine.benbettaieb@u-bourgogne.fr (N.B.); ali.assifaoui@u-bourgogne.fr (A.A.)

Figure S1: SEM images and elementary analysis

SEM images of clays at the surface of the composite film. The elementary analysis concerns the red square.

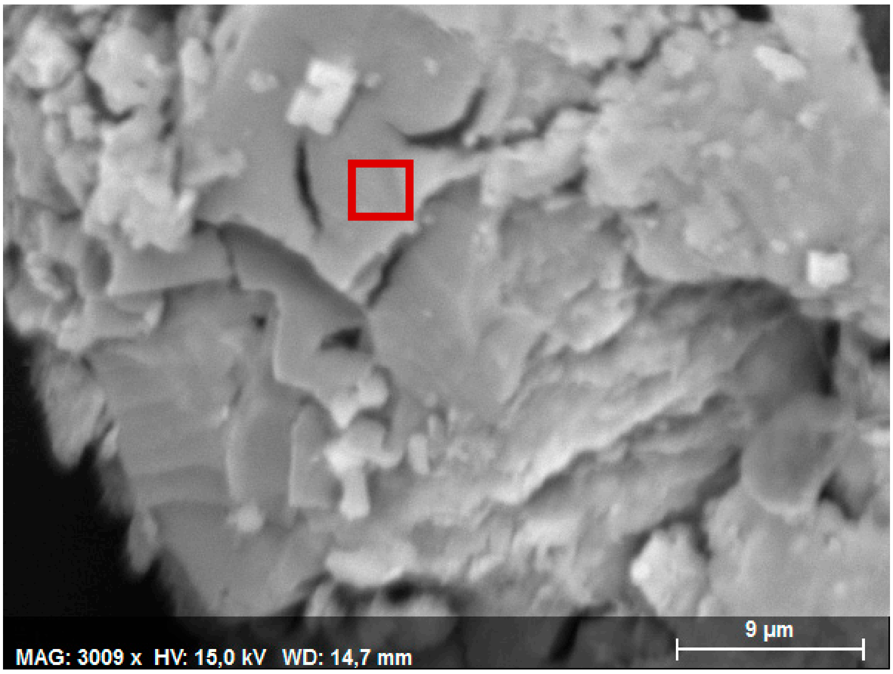

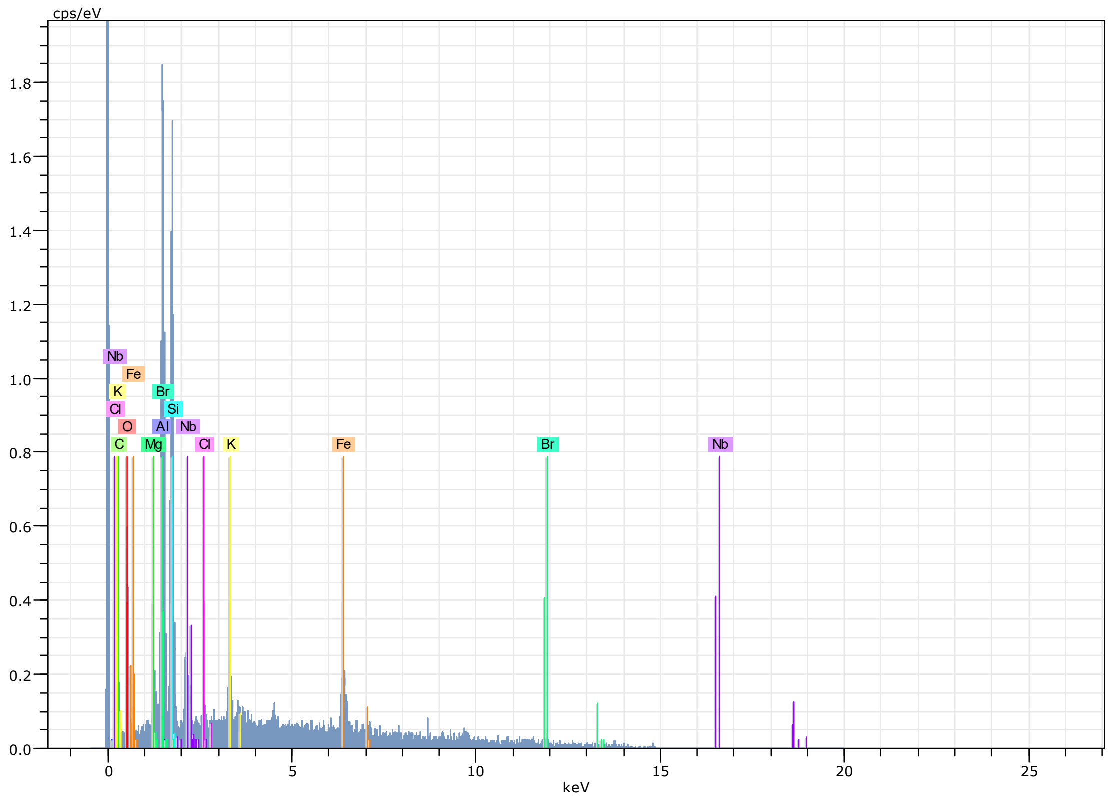

Spectrum: C5OM5

El	AN	Series	unn. C [wt.%]	norm. C [wt.%]	Atom. C [at.%]	Error (1 Sigma) [wt.%]
C	6	K-series	17,63	20,08	41,82	5,00
O	8	K-series	12,96	14,76	23,07	3,11
Mg	12	K-series	0,61	0,70	0,72	0,10
**Al**	**13**	**K-series**	**2,32**	**2,65**	2,45	0,19
**Si**	**14**	**K-series**	**17,30**	19,71	17,55	0,84
Cl	17	K-series	0,36	0,41	0,29	0,07
**K**	**19**	**K-series**	**2,00**	**2,28**	**1,46**	**0,14**
**Fe**	**26**	**K-series**	**3,37**	**3,84**	1,72	0,21
**Br**	**35**	**L-series**	**26,66**	**30,36**	9,51	1,40
Nb	41	L-series	4,58	5,21	1,40	0,29
		Total:	87,80	100,00	100,00	

The elementary analysis showed the presence of Al, Si, Br, Fe which are the main element present in the modified clay. Such result indicates clearly the presence of the clay at the surface of the clay. The high amount of C is due mainly to the coating which was made to increase the resolution of the images

Figure S2: GC-MS of the linseed oil

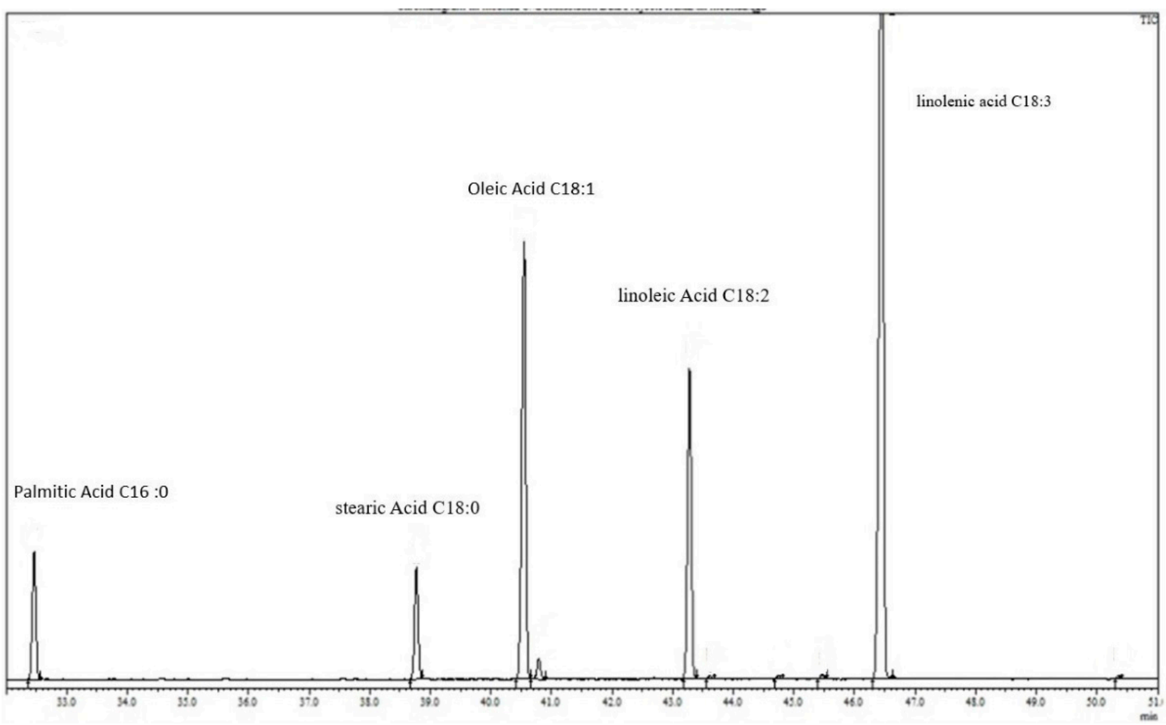

Carbon length	Fatty Acids	FA composition (%)
	Saturated FAs	
C16:0	Palmitic Acid	5.87
C18:0	Stéaric Acid	5.15
C20:0	Arachidic Acid	0.12
C22:0	Béhénic Acid	0.1
	∑ saturated FAs	11.24
	Monounsaturated FAs	
C18:1Δ9c	Oleic Acid	22.04
C18:1Δ11c	Vaccenic Acid	0.98

	∑ mono_unsaturated FAs	23.02
	Polyunsatured FAs	
C18:2Δ9.12	linoleic Acid ω6	14.90
C18:2Δ11.14	11.14 octadecadienoic Acid	0.10
C20:3Δ11. 14.17	Eicosatrienoic	0.20
C18:3Δ9.12.15	α linolenic Acid ω3	50.46
∑ Poly-unsaturated FAs		65.66
Unsaturated /saturated		7.88
ω3/ ω6		3.38
